# Identification of CFHR4 associated with poor prognosis of hepatocellular carcinoma

**DOI:** 10.3389/fonc.2022.812663

**Published:** 2022-10-20

**Authors:** Qinglin Ding, Hanluo Li, Zhigao Xu, Kanghong Hu, Qifa Ye

**Affiliations:** ^1^Sino-German Biomedical Center, National Center for Cellular Regulation and Molecular Pharmaceutics, Cooperative Innovation Center of Industrial Fermentation (Ministry of Education & Hubei Province), Hubei University of Technology, Wuhan, China; ^2^Institute of Hepatobiliary Diseases of Wuhan University, National Quality Control Center for Donated Organ Procurement, Hubei Key Laboratory of Medical Technology on Transplantation, Zhongnan Hospital of Wuhan University, Wuhan, China

**Keywords:** complement factor H-related protein 4 (CFHR4), hepatocellular carcinoma (HCC), immune infiltration, biomarker, prognosis

## Abstract

**Background:**

Hepatocellular carcinoma (HCC) is one of the most leading causes of cancer death worldwide. The 5-year survival rate of HCC patients remains low due to the lack of early-stage symptoms. Human complement factor H-related protein 4 (CFHR4) is a critical gene that belongs to the factor H family of plasma glycoproteins, which has not been linked to HCC development. The correlations between CFHR4 and prognosis and tumor-infiltrating lymphocytes in HCC are yet unknown. The present study demonstrated the involvement of CFHR4 in HCC *via* data mining approaches.

**Results:**

A total of 18 upregulated and 67 down-regulated differentially expressed genes (DEGs) were identified. Importantly, CFHR4, which was screened from DEGs, was shown to express at a lower level in HCC tumor tissue than normal tissues. Western blotting (WB), immunohistochemical (IHC) and quantitative reverse transcription PCR (qRT-PCR) experiments of clinical samples further validated CFHR4 was aberrantly expressed in HCC patients; Data from TCGA showed that CFHR4 was inversely correlated with a cancer family history, histological grade, tumor node metastasis (TNM) stage, and serum AFP level of HCC patients; Univariate and multivariate analyses revealed that low expression of CFHR4 was an independent predictive marker in patients with HCC; Kaplan-Meier analysis showed that the lower expression of CFHR4 was significantly associated with the progression of HCC and poor prognosis rates. Furthermore, TIMER analysis indicated that CFHR4 expression levels had correlations with infiltrating levels of immune cells in HCC.

**Conclusion:**

CFHR4 expression was low in HCC and was significantly related to the poor prognosis of HCC and the level of immune infiltration. CFHR4 played important roles in regulating the initiation and progression of HCC and could be a potential biomarker for the diagnosis and prognosis of HCC.

**Methods:**

The expression of CFHR4 was analyzed by GEO and TCGA-LIHC database and verified by WB and IHC assay. The biological function of CFHR4 was performed by GO and KEGG enrichment analysis, and the genomic alteration of CFHR4 was investigated by cBioPortal database.The correlation between CFHR4 expression and clinical relevance was evaluated through Cox proportional hazards model, and the correlation between CFHR4 expression and tumor immune infiltrates were studied by TIMER database.

## Background

Hepatocellular carcinoma (HCC) is one of the most lethal causes of cancer deaths worldwide, of which the incidence in China stays the highest globally. HCC is also the second in the mortality of malignant tumors in China with the five-year survival rate only 10% (1, 2). Unfortunately, lacking of typical symptoms in the early stage of carcinogenesis, majority of primary HCC patients is generally diagnosed at advanced stage once detected, with hepatitis, cirrhosis and other severe clinical symptoms (3). Despite of compelling advancement in the early detection, surgical intervention, and comprehensive therapy for HCC, the clinical prognosis for HCC remains poor (4). Compared with other cancers, non-surgical interventions for HCC including primary and second-line drugs are limited in clinical practice with low availability and poor clinical efficacy. An accurate diagnosis assessment is critical in the prognosis and therapeutic treatments, and thereby live-saving for a great many of HCC patients (5). It is crucial to discover and identify novel diagnostic and prognostic biomarkers with improved sensitivity, accuracy, and specificity on HCC (6). Extensive efforts using high-throughput transcriptome sequencing and bioinformatics techniques reveal the potential gene targets of prognostic significance in HCC, and provide a reference for identifying and investigating novel biomarker for HCC.

One critical limitation on identifying specific biomarkers and efficient treatments for HCC is the tumor microenvironment (TME), which is an important determinant on the tumorigenesis, progression, malignancy, recurrence and drug resistance of HCC (7). TME involves a complex organization of cancer cells, stromal cells, cytokines and extracellular matrix. As an inflammation-related cancer, HCC progresses in a specific immunosuppressive TME, with intensive immune infiltration of TME stromal cells including tumor-associated macrophages (TAMs), tumor-associated neutrophils (TANs) and myeloid-derived suppressor cells (MDSCs), regulatory T cells (Tregs) and dendritic cells (DCs) (8, 9). By interfering these tumor associated immune, HCC interact with the TME to achieve immune-excluded phenotypes for immune escape and tumor malignancy.

The complement system is a primary mechanism of innate immunity, consisting of 50 plasma-soluble and membrane-expressed proteins, which recognizes the invading pathogens from the host tissue, complements and augments the antibody-driven phagocytosis after activation (10). There are 3 pathways of complement activation, including classical pathway, alternative pathway (AP) and lectin pathway, all of which initiate the production of protease C3-convertase and C5-convertase, activate complement cascades complement component 5 (C5) and form C5b-9 membrane attack complex (MAC), which leads to the antibody-mediated complement-dependent cytotoxicity (CDC) (11). AP is a direct and rapid complement pathway, which was triggered by the natural hydrolysis of C3 protein and direct binding of C3b to the microbes (12).

Recent studies have shown immune-regulatory effects of complement activation in the TME and tumor progression. It was found that there are increased production of complement proteins and maladjusted complement components in the tumor and TME (13). Beside the healthy host cells, cancer cells also produce complement proteins, e.g. in the clinical cases of melanoma and breast, colon, lung, and pancreatic cancer, as well as animal models of breast, cervical and ovarian cancer (14–19). The pathologically production and activation of complement proteins in the TME promotes the tumor progression, yet the main pathway of complement activation in the TME remains unclear (20). Cancer cells generate complement proteins C1q, C3a and C5a, which increase tumor cell proliferation through enhanced ERK1/2, SAPK/JNK, and p38 phosphorylation, and facilitate the tumor progression with immune-suppressive TME (21–23). Moreover, the interaction of complement system and TME stromal cells promotes the tumor progression and malignance. Activation of complement proteins in TME can recruit TAMs, TANs and MDSCs to the tumor sites, polarize them towards the tumor-promoting phenotypes, which enhance tumor growth and metastasis (24, 25).

Among the complement proteins, complement factor H (FH) is the main down-regulator of the AP pathway. By competitive binding to C3b against the factor Bb and inhibit the formation of the C3 convertase, FH maintains the low density of C3b molecules and protect the host cells from the complement-mediated damage (26). FH-related proteins (FHRs) are evolutionarily and structurally related to FH, and intensively associated with various complement disorders and tumor progression (20, 27). Many complicated genetic disorders are caused by variations in the factor H gene family (28).

Complement factor H-related protein 4 (CFHR4) is a variant of FHRs, and its gene is located on the human 1q32 chromosome (29) and consists of 10 coding exons (30). The CFHR4 cDNA isolated from the human liver cDNA library is 1315bp in length and encodes an open reading frame of 331 amino acids (31). By alternative splicing, CFHR4 obtained two different mRNA transcripts CFHR4A and CFHR4B (30). As an amphiphilic protein, CFHR4 exists in human plasma and triglyceride-rich lipoproteins in the monomer or dimer form (31, 32) and can play a role as a component of lipoproteins (33). CFHR4 consists of a short common repetitive sequence (SCR) domain, which binds C-reactive protein through its N-terminal SCR, activates the classical complement pathway, regulates complement-mediated foreign antigen processing (34), and enhances the regulation on phagocytosis of microorganisms (35–37). As an important complement protein and immune regulator, CFHR4 is a plasma glycoprotein in the complement factor H family, involved in various immune disorders. Studies have shown that CFHR4 shows abnormalities in various diseases, which may be connected to disease incidence and progression. For instance, the absence of CFHR4 increases the risk of the atypical hemolytic uremic syndrome (38, 39), and the increase of circulation level of CFHR4 was strongly associated with age-related macular degeneration (AMD) (34, 40). Deletion of CFHR4 may limit the ability of C protein to inhibit inflammation, thus promoting the development of systemic lupus erythematosus (SLE) (29, 39).

Currently, the expression of CFHR4, engaged in the aforementioned immune responses and tumor progression, especially in HCC has not yet been investigated (41). It is intriguing to investigate the altered expression level of CFHR4 in HCC and adjacent tissues, which might lead to a discovery of a potential HCC biomarker for the diagnosis and/or prognosis.

In this study, bioinformatic analysis was performed to address the correlation of CFHR4 with HCC, and the expression of CFHR4 in GEO and TCGA databases was assessed, in parallel with the expression of CFHR4 in HCC specimens *via* Western Blotting and immunohistochemistry (5, 41). Meanwhile, genetic alterations of CFHR4 were analyzed by the cBio for Cancer Genomics Portal (cBioPortal) database, and immune cell enrichments in HCC were also analyzed by Tumor IMmune Estimation Resource (TIMER) database (42). Furthermore, the CFHR4 expression on the survival for HCC patients was also assessed by Kaplan–Meier plotter. Thus, this study aimed to evaluate the potential role of CFHR4 to be a biomarker for HCC diagnosis and/or prognosis, with the expectation of providing novel insights into the tumorigenesis and progression of HCC (5, 43).

## Results

### The identification of DEGs in HCC

**Figure 1:**
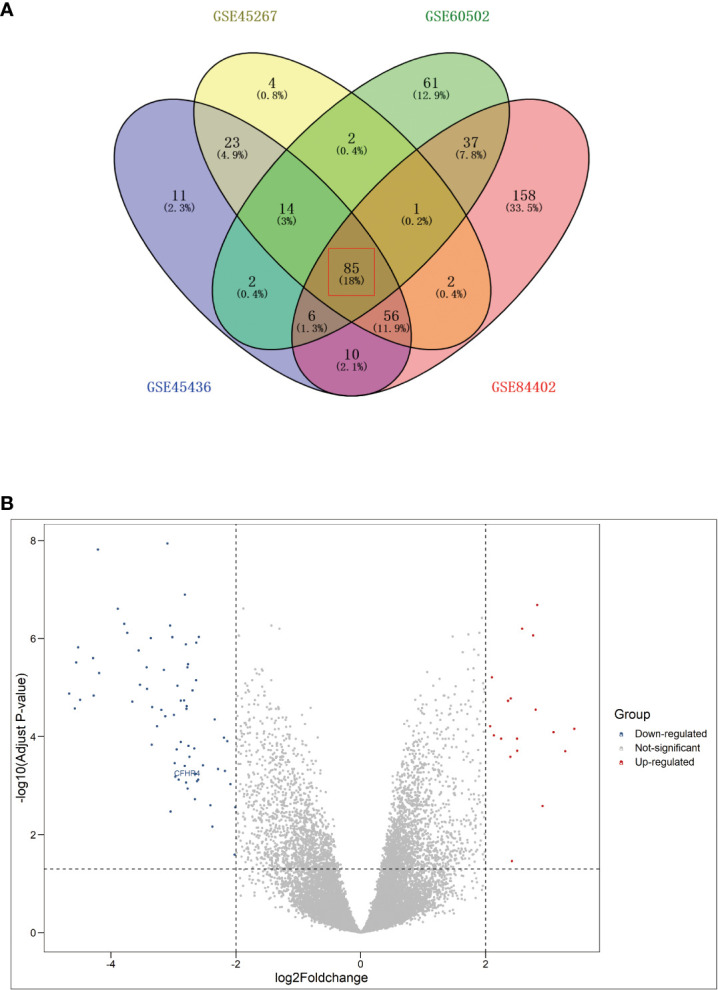
Analysis of differentially expressed genes (DEGs) in 4 GSE Series. **(A)** Venn diagram of DEGs (|logFC| >2, P < 0.05).); **(B)** Volcano plot of DEGs.

DEGs between HCC tumor and non-tumor samples were investigated on the GEO dataset (GSE45267, GSE45436, GSE60502, and GSE84402) by applying bioinformatics analysis (**Table 1**). In total, 18 significantly upregulated genes and 67 significantly down-regulated genes were identified (including CFHR4), as listed in **Figure 1** and **Table 2** (44, 45).

**Table 1:** Details of the GEO series included in this analysis.

GEO series	Contributor(s), Year	Number of samples (Tumor/Control)	Platform
GSE45267	Chen CL, et al., 2018	87 (46/41)	[HG-U133_Plus_2] Affymetrix Human Genome U133 Plus 2.0 Array.
GSE45436	Wang HW, et al., 2013	134 (41/93)	[HG-U133_Plus_2] Affymetrix Human Genome U133 Plus 2.0 Array.
GSE60502	Kao KJ., 2014	36 (18/18)	[HG-U133A] Affymetrix Human Genome U133A Array.
GSE84402	Wang H, et al., 2017	28 (14/14)	[HG-U133_Plus_2] Affymetrix Human Genome U133 Plus 2.0 Array.

**Table 2:** 85 Differentially expressed genes (DEGs) were identified from four GEO datasets.

DEGs	Number	Gene names
Up-regulated	18	MDK ASPM KIF20A HMMR MELK NCAPG PBK TOP2A CCNB1 CDC20 NDC80 PEG10 RRM2 PRC1 TTK GPC3 GINS1 SPINK1
Down-regulated	67	HAMP C9 FCN3 MT1M SLC22A1 CLEC1B CYP1A2 CRHBP NAT2 SLCO1B3 APOF GYS2 GBA3 HPD MT1F CYP2A6 CFHR4 LPA AFM ADH1C C6 CYP26A1 AKR1D1 FOS CXCL14 CLEC4M KCNN2 HGFAC MT1X PCK1 MARCO SLC10A1 MT1H SRPX GLYAT LECT2 CD5L HSD11B1 FETUB MT1E SPP2 CETP RDH16 ADH1B DNASE1L3 KMO IGF1 GNMT CFP SRD5A2 C8A CYP2B6 BCHE CYP2C9 CYP39A1 MT1G CYP2A7 LYVE1 FCN2 NPY1R GSTZ1 CYP3A4 ZG16 FOSB C7 CYP1A1 OTC

### The comparison of CFHR4 expression

**Figure 2:**
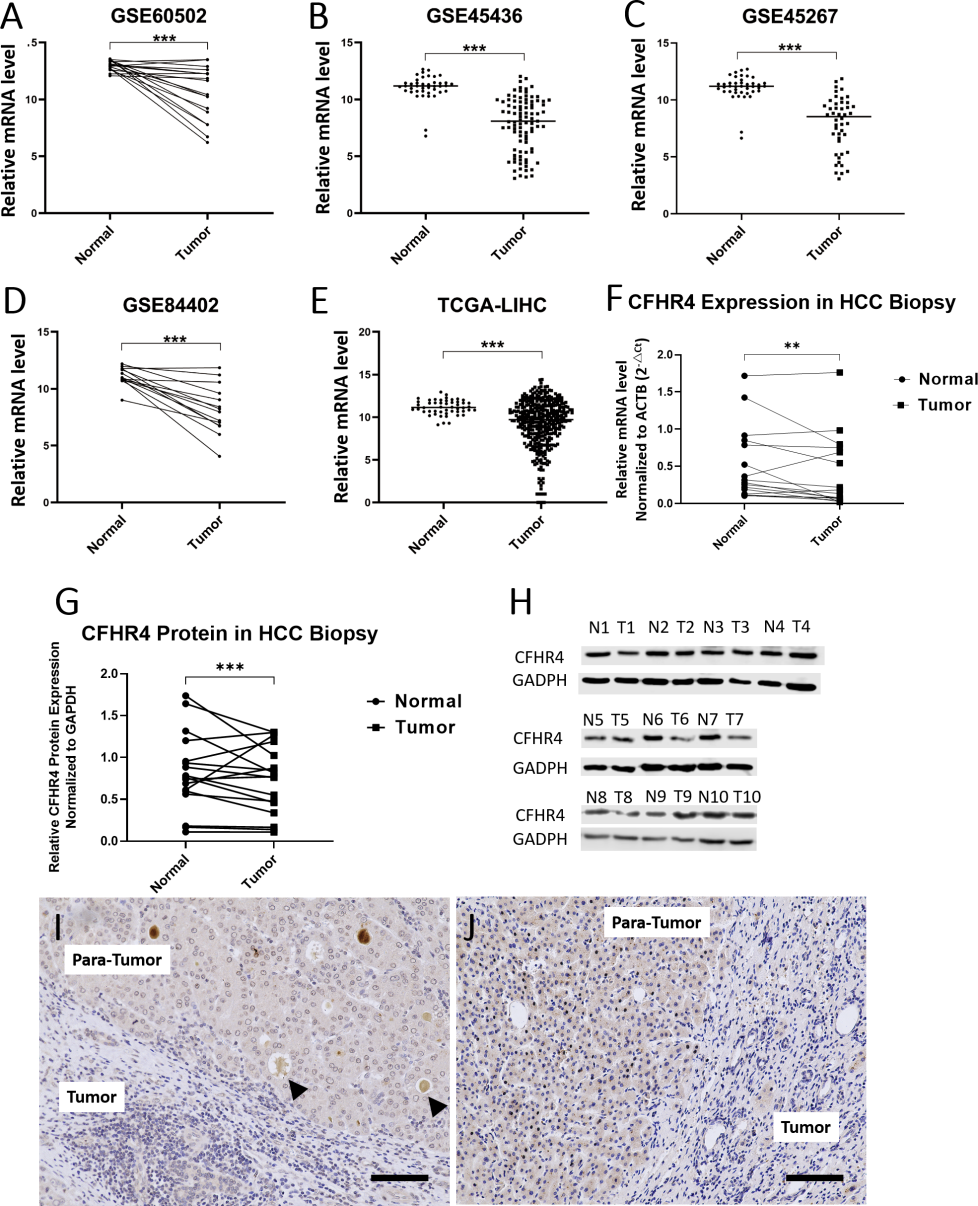
CFHR4 expression levels between tumor and non-tumor tissues in HCC patients. **(A–E)** Analysis of mRNA transcriptional level of CFHR4 in normal and tumor tissues using data from the GEO and TCGA-LINC database; **(F, G)** Comparisons on the mRNA and protein levels of CFHR4 between tumor and non-tumor tissues from 17 patients; **(H)** Western Blotting was used to analyze and compare the expression of CFGR4 protein in 10 HCC tissues and adjacent non-tumor tissues, respectively GAPDH was corrected as an internal reference. **(I, J)** Immunohistochemical staining of CFHR4 on the HCC and para-tumor tissue. “T” represents the HCC tissues, and “N” the non- tumor tissues. The same number after “N” and “T” indicates the same patient. Black arrows represent the dissociative CFHR4 secreted into circulation through intrahepatic veins. Scale: 100 μm, *P* < 0.01 **, *P* < 0.001 ***.

The mRNA transcription of CFHR4 in tumor samples was significantly lower than non-tumor samples in GSE45267, GSE45436, GSE60502, GSE84402, and TCGA-LIHC dataset, as indicated in **Figures 2A–E**. To analyze the levels of gene and protein expression of CFHR4 in the HCC and adjacent non-tumor tissue, tissue biopsies were obtained from 17 HCC patients who underwent HCC excision surgeries in the Institute of Hepatobiliary Diseases of Zhongnan Hospital, Wuhan University (**Table 3**). All the experiments were institutional approved by Hubei University of Technology and Zhongnan Hospital of Wuhan University, and performed with the ethical guidance.

**Table 3:** Clinical information of 17 HCC cases for obtaining biopsies for validating the CFHR4 expression.

Variables	No. Patients (n = 17)
Age	
<55	10
≥55	7
Gender	
Male	14
Female	3
Liver Cirrhosis	
None	5
Mild	4
Severe	8
Fatty Liver	
None	17
HBV	
Positive	9
Negative	8
No. HCC Primary Tumor	
≥3	1
<3	16
Cumulative Size of Primary Tumor	
≤8 cm	7
>8 cm	10
Preoperative AFP	
≤400 ng/ml	4
>400 ng/ml	13
Macrovascular Invasion	
Yes	2
No	15
Portal Vein Tumor Thrombus	
Yes	6
No	11
Intrahepatic Metastasis	
Yes	3
No	14
TNM Staging	
I-II	6
III-IV	11
CFHR4 Expression	
Positive	17

Results of qRT-PCR revealed that the mRNA level of CFHR4 was significantly lower in the HCC tissue than that in the non-tumor tissue (*P*<0.0001), as shown in **Figure 2F**. Western blot displayed correlated results that the protein content of CFHR4 was higher in the normal non-tumor tissue with significance (*P*<0.001), in **Figures 2G, H**. The immunohistochemistry (IHC) analysis showed relatively intensive staining of CFHR4 antibody in the normal hepatocyte tissue. In contrast, large amount of HCC cells expressed lower level of CFHR4 protein (**Figures 2I, J**). Interestingly, there was intensive CFHR4 deposition in the terminal vein of the healthy para-tumor tissue, indicating that the complement factor CFHR4 is produced by hepatocyte and secreted into circulation through intrahepatic veins (black arrows in **Figures 2I, J**, scale bar 100μm).

### The correlation and GO/KEGG enrichment analysis results

**Figure 3:**
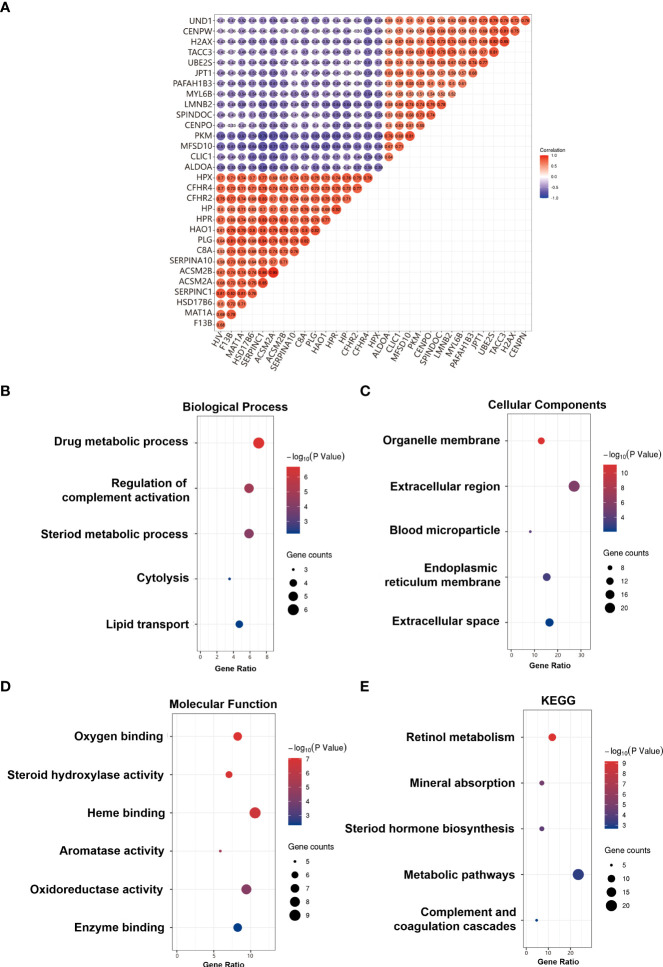
Correlation and GO/KEGG enrichment analysis results. **(A)** Heat map of positive and negative correlation genes with CFHR4 (Top 10). **(B)** GO_BP enrichment results. **(C)** GO_CC enrichment results. **(D)** GO_MF enrichment results. **(E)** KEGG enrichment results.

The correlation between CFHR4 and CFHR4-correlated genes was identified (**Figure 3A**). GO analysis results proved that CFHR4-correlated genes were significantly enriched in the drug-metabolic process, regulation of complement activation, steroid metabolic process, cytolysis, lipid transport at the Biological Process (BP) levels; organelle membrane, extracellular region, blood microparticle, endoplasmic reticulum membrane, extracellular space at the Cellular Component (CC) levels, and oxygen binding, steroid hydroxylase activity, heme binding, aromatase activity, oxidoreductase activity, enzyme binding at the Molecular Function (MF) levels. Additionally, the KEGG pathway enrichment of CFHR4 interactive genes showed that retinol metabolism, mineral absorption, steroid hormone biosynthesis, metabolic pathways, complement components, and coagulation cascades, were the enriched pathways (5) (**Figures 3B–E**).

### The genomic mutation of CFHR4

**Figure 4:**
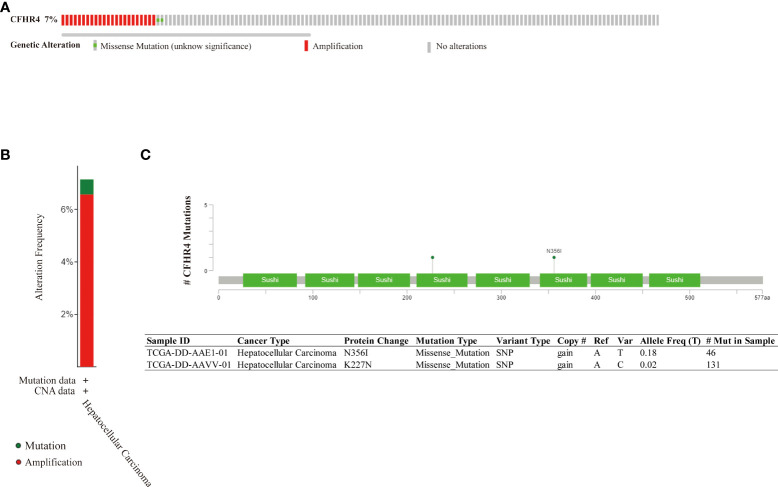
Genomic mutation of CFHR4 in HCC. **(A)** The genetic alterations of CFHR4. **(B)** The genetic alteration frequency of CFHR4. **(C)** The missense mutation of CFHR4 amino acids.

The genomic mutation is closely associated with tumorigenesis. The research showed about 7% of genetic alteration of CFHR4 in HCC, including amplification and missense mutation with unknown significance (**Figures 4A, B**). In addition, missense mutation of CFHR4 resulted in the amino acid change, including asparagine (N) 356 replaced by isoleucine (I) and lysine (K) 227 replaced by asparagine (N) (**Figure 4C**). The above results indicated that genetic alteration of CFHR4 could be found in HCC, which might play an important role in tumorigenesis of HCC (42).

### The correlation of CFHR4 expression with the clinicopathological characteristics of HCC

Chi-square test was used to analyze the correlation between CFHR4 expression and clinicopathological factors (**Table 4**). Studies have shown that low CFHR4 expression was significantly associated with age (*P* = 0. 026), BMI (*P* = 0.013), race (*P <*0.001), and family history of cancer (*P* = 0.001), histological grade (*P <*0.001), TNM stage (*P* = 0. 048), and serum AFP level (*P <*0.001) (26-28). (*P* < 0.05 *, *P* < 0.01 **, *P* < 0.001 ***)

**Table 4:** The characteristics of HCC patients between CFHR4 high and low groups.

Variables	CFHR4 expression level		*P* value
	Low (n = 180)	High (n = 180)	
Age			0.026*
<55	66	47	
≥55	113	134	
Gender			0.681
Male	119	124	
Female	60	57	
BMI			0.013*
<18.5	12	9	
18.5~24.99	91	61	
25-29.99	35	52	
>30	29	38	
Race			<0.001***
Asian	103	52	
White	68	108	
Black or African American	5	12	
Family history of cancer			0.001**
No	113	89	
Yes	40	69	
Histological grade			<0.001***
G1-G2	95	129	
G3-G4	84	47	
TNM stage			0.048*
I-II	119	129	
III-IV	53	35	
AFP (ng/ml)			<0.001***
>400	44	19	
≤400	85	122	
Hepatic inflammation			0.056
None	42	72	
Mild	51	46	
Severe	6	11	

360 clinical cases were analyzed from the dataset database “TCGA-LIHC” from GDC database.

*P<0.05, **P<0.01, ***P<0.001.

### The independent prognostic value of CFHR4 expression

**Figure 5:**
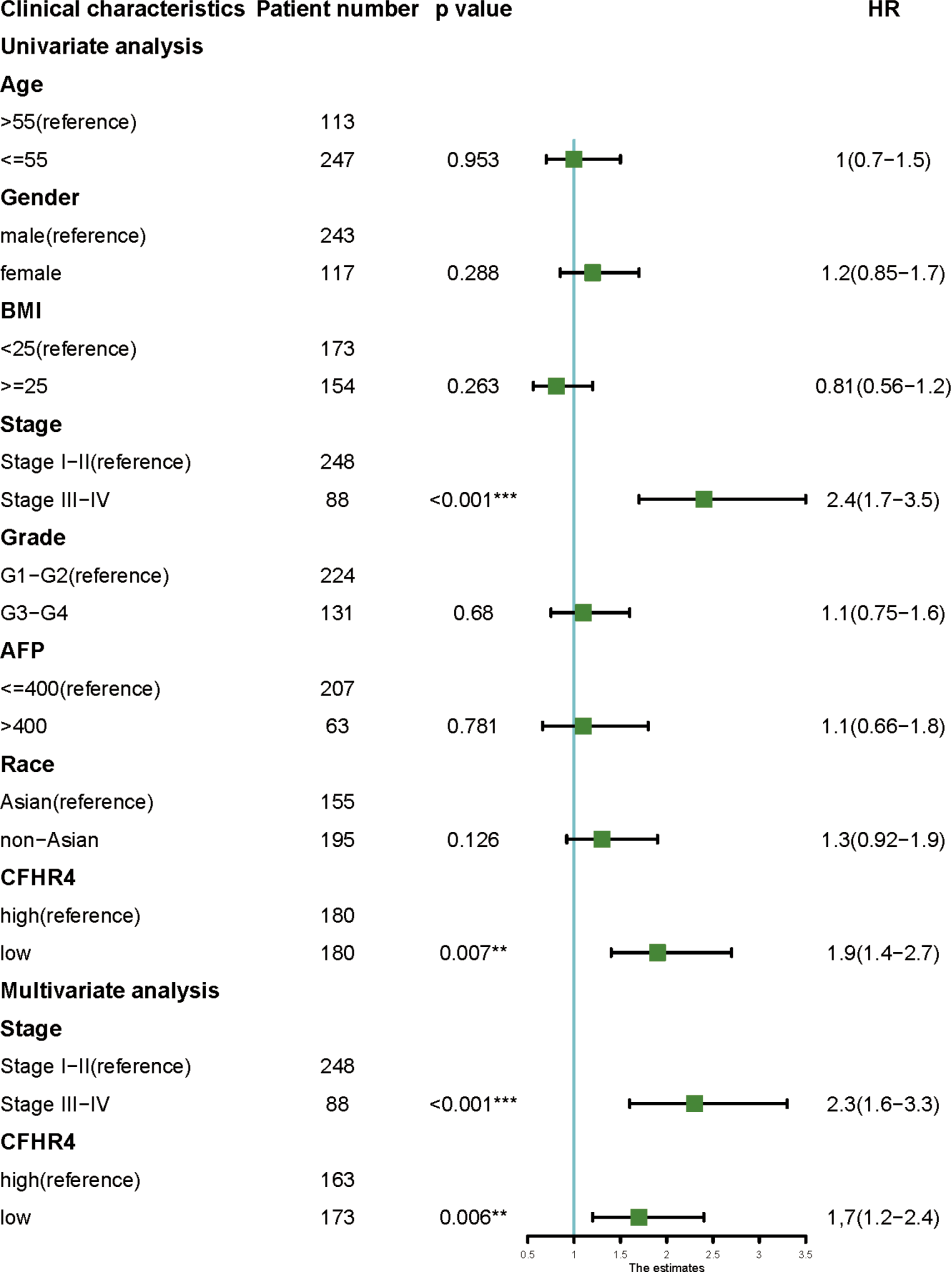
Univariate and multivariate analyses of clinicopathological factors for OS in HCC. *P* < 0.01 **, *P* < 0.001 ***.

The univariate analysis indicated that CFHR4 expression and TNM stage were directly correlated with OS in HCC. Furthermore, by Cox multivariate analysis, we found that low CFHR4 expression and TNM stage were confirmed as independent prognostic indicators influencing the prognosis of HCC patients (**Figure 5**) (42).

### The association between CFHR4 and survival

**Figure 6:**
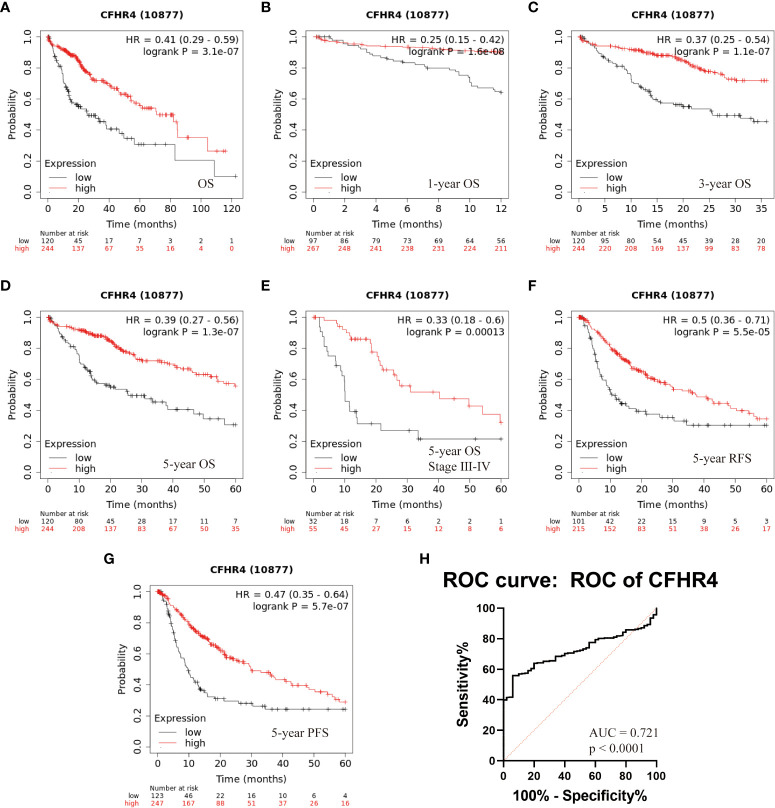
Kaplan-Meier survival analysis of HCC patients with respect to CFHR4 expression. **(A–D)** The association between CFHR4 expression and Overall survival (OS). **(E)** Multivariate analyses of TNM stage and CFHR4 expression with the 5-year OS. **(F, G)** The associations between CFHR4 expression and 5-year relapse free survival (RFS), as well as CFHR4 and 5-year progression free survival (PFS). **(H)** The assessment of receiver operating characteristic (ROC) curve for CFHR4 expression.

The results outlined that low expression of CFHR4 in tumor tissues was considerably associated with poor overall survival (OS, log rank *P* = 3.1e-07, HR = 0.41 (0.29-0.59), **Figure 6A**) in patients with HCC. Moreover, a subgroup analysis revealed that the down-regulation of CFHR4 in tumor tissue was a risk factor for reduced 1-year OS (log rank *P* = 1.6e-08, HR = 0.25 (0.15 -0.42), **Figure 6B**), 3-year OS (log rank *P* = 1.1e-07, HR = 0.37 (0.25 -0.54), **Figure 6C**), 5-year OS (log rank *P* = 1.3e-07, HR = 0.39 (0.27 -0.56), **Figure 6D**), 5-year OS stage III-IV (log rank *P* = 0.00013, HR = 0.33 (0.18-0.60), **Figure 6E**), 5-year relapse-free survival (RFS, log rank *P* = 5.5e-05, HR = 0.5 (0.36-0.71), **Figure 6F**), and 5-year progression free survival (PFS, log rank *P* = 5.7e-07, HR = 0.47 (0.35-0.64), **Figure 6G**) in HCC patients (5).

### The diagnostic performance of CFHR4 mRNA Levels for the differentiation of HCC patients from healthy controls

The potential diagnostic utility of CFHR4 to differentiate between HCC and benign disease was assessed through generating a receiver operating characteristic (ROC) curve for CFHR4 expression. We found that the ROC area under the curve (AUC) of the CFHR4 was 0.72 (95% confidence interval (CI): 0.67–0.77) (**Figure 6H**). However, we do not have a serum sample to verify our point, and considerable work needs to be done.

### The correlation analysis between CFHR4 expression and tumor-infiltrating immune cells

**Figure 7:**
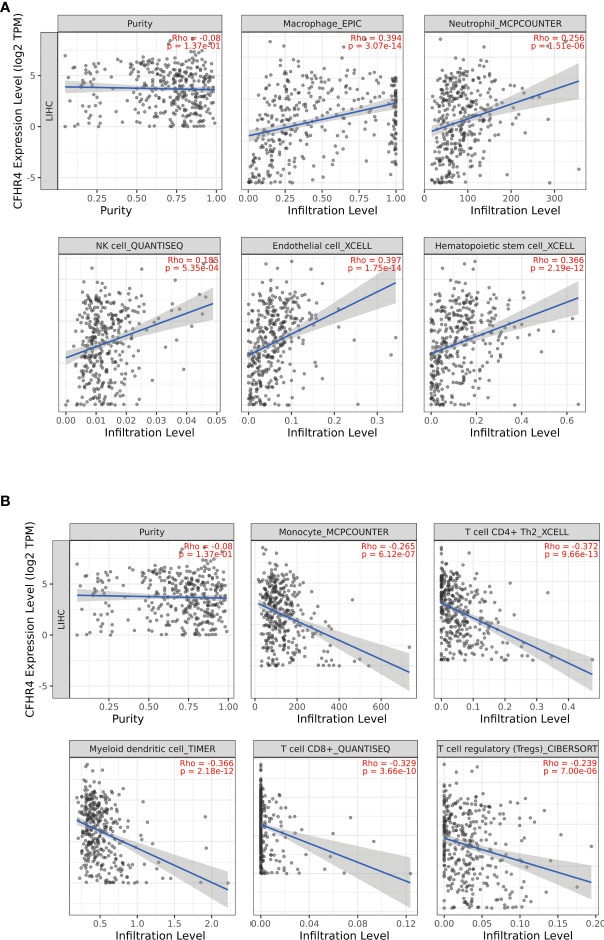
Correlations of CFHR4 expression with tumor-infiltrating immune (TII) level in HCC. **(A)** The correlation between CFHR4 expression and the infiltrating levels of macrophages cells, neutrophils cells, NK cells, endothelial cells and Hematopoietic stem cells in LIHC. **(B)** The correlation between CFHR4 expression and the infiltrating levels of monocytes cells, CD4+ T cells, myeloid dendritic cells, CD8+ T cells, and regulatory T cells (Tregs) in LIHC.

We analyzed the correlation between CFHR4 expression and infiltrating immune cells (monocyte, CD4+ T cells, myeloid dendritic cells, CD8+ T cells, T cells regulatory (Tregs), macrophages, neutrophils, NK cells, endothelial cells and hematopoietic stem cells). The results showed that the expression of CFHR4 was positively correlated with the infiltration levels of macrophages (r = 0.394, *P* = 3.07e-14), neutrophils (r = 0.256, *P* = 1.51e-06), NK cells (r = 0.385, *P* =5.35e-04), endothelial cells (r = 0.397, *P* =1.75e-14), and hematopoietic stem cells (r = 0.366, *P* = 2.19 e-12) (**Figure 7A**), and negatively correlated with monocytes (r = -0.265, *P* = 6.12e-07), CD4+ T cells (r = -0.372, *P* = 9.66e-13), myeloid dendritic cells (r = -0.366, *P* =2.18e-12), CD8+ T cells (r = -0.329, *P* =3.66e-10), and regulatory T cells (Tregs) (r = -0.239, *P* = 7.00 e-06) (**Figure 7B**) (28).

## Discussion

Complement system serves as an essential elements in innate immunity, which is also in depth engages in the tumor progression and TME regulation. The liver synthesizes and secrets most of the complement components, and it is important to investigate and elucidate the differential expression profiles of complement proteins in the normal liver tissues and malignant HCC tumors.

This study is the first to report and relate the expression profile of CFHR4 with the clinical progression of HCC. It was found that the mRNA level of CFHR4 in HCC was significantly lower than that in the adjacent normal liver tissues. CFHR4 mRNA levels were inversely correlated with a cancer family history, histological grade, TNM stage, and serum AFP level of HCC patients. The results of the high-throughput data comparisons were validated using clinical biopsies from 17 HCC patients, which support the bioinformatics analysis (*P*<0.001).

The down-regulation of CFHR4 in HCC cells might facilitate the tumor progression and malignancy. One supportive evidence is CFHR3, a similar variant of CFHR4 with overlapping functions, was reported highly expressed at the normal liver tissue, while the HCC tumor expressed CFHR3 in significantly lower level. HCC patients with high level of CFHR3 expression showed improved overall survive (46). Over-expression of CFHR3 in the HCC cells induced cell apoptosis by activating Bax and Caspase−3, and inhibited cell proliferation by decreasing Ki67, Bcl−2 and surviving (46). This study underpins the findings that, the down-regulation of CFHR expression in HCC cells may promote the cancer proliferation and tumor progression by exempting the inhibition of PI3K/Akt/mTOR pathway (46).

On the other hand, by analyzing public datasets, we have observed the correlation between the level of intracellular CFHR4 mRNA and status of infiltrating immune cells in HCC. We speculate that the expression of CFHR4 in HCC may be an important mediator for the development of TME, which involves in the tumor immune evasion.

As a negative regulator of AP pathway, the production of FH in cancer cells generates a tumor-established complement inhibition threshold and protects tumor cells from the CDC attack, which promote tumor immune evasion (47). FH also plays a crucial role in interacting with the TME cells. It was found that FH and C1q interact with complement PTX3 to activate the complement cascade, resulting in the recruitment of TAM with increased macrophage infiltration, cytokine production, and angiogenesis (48). FH produced by the DCs in the TME inhibits the proliferation of CD4+ T cells, thereby creates an immunosuppressive TME that enhance the tumor progression and radiotherapy-resistance (49, 50).

Moreover, it was reported that complement proteins C7 and FH maintain the stemness of patient-derived and immortalized HCC cell lines *via* phosphoglucan phosphatase LSF-1 dependent mechanism, and were significantly up-regulated in the tumor sphere *in vivo* (51). Knockdown of FH expression in cutaneous squamous cell carcinoma suppressed the cell proliferation and migration through inhibited ERK1/2 and p38 signaling (52). Increasing evidences on the expression of FH and cancer cell stemness may, therefore, imply the relevance of FH differentiation degree of HCC.

Unlike FH, a suppressor of complement AP, CFHR4 is considered as an enhancer of AP. CFHR4 is an antagonist of FH and they competitively binds to the C3b and Bb. The consequent CFHR4-C3bBb convertase is more resistant to the factor H-mediated decay (35). It was found that CFHR4 competed and deregulated FH on the surface of tumor cells, which efficiently led to the macrophages phagocytosis through CDC attack (36). Therefore, it is reasonable to speculate that the over-expression of CFHR4 within the tumor might allow the complement system to reverse the immune suppressive phenotype cause by the FH, thus overcome the immune escape ultimately. Decreased CFHR4 in HCC might be relevant with resistance of tumor cells against the mechanism of CFHR4-enhanced complement attack as mentioned, leading to the disordered immune regulation and tumor survival from the complement-mediated attack. These results suggest that CFHR4 plays a potential role in the HCC occurrence and development, and may represent a novel biomarker for the tumor diagnosis and/or prognosis, as well as therapeutic target.

Obviously, some of the findings were based on bioinformatics data analysis, which require further experimental validation in various HCC models. In the future study, the tumorigenesis, progression, TME contents and level of complement immunity should be elaborately conducted using over-expression and under-expression of CFHR4 in both *in vivo* and *in vitro* models. To explore the underlying mechanism of specific inhibition of CFHR4 on the activity of tumor-infiltrating lymphocytes and the anti-tumor efficacy of CFHR4 expression represent an interesting topic for future study. Moreover, CFHR4 is a complement factor produced by the liver and secreted into circulation. Whether abnormal alteration of CFHR4 content in blood reflects the tumorigenesis and progression of HCC remains to be investigated using a larger number of clinical samples.

## Conclusion

In conclusion, this study discovers that the CFHR4 expression is down-regulated in the HCC tumors and correlates with the infiltration of TME associated cells in HCC to a certain extent. The association between CFHR4 and several physiopathological characteristics in HCC suggests that it might be a possible predictive indicator for HCC, implying that CFHR4 may be a potential immune biomarker of HCC. This work contributes to a better understanding of the possible role and prognostic significance of CFHR4 in tumor immunology.

## Materials and methods

### Data resource and description

Four GEO microarray series (GSE45267, GSE45436, GSE60502, GSE84402) containing HCC tumor and normal samples were downloaded from the National Center for Biotechnology Information’s (NCBI) Gene Expression Omnibus (GEO, https://www.ncbi.nlm.nih.gov/geo/). Platforms and samples of the GEO series were summarized in **Table 2** (5).

Clinical information of HCC patients were obtained from the GDC database with the project ID “TCGA-LIHC”, dbGaP Study Accession: phs000178 (https://portal.gdc.cancer.gov/projects/TCGA-LIHC). Amid these, 360 clinical cases with RNA-Seq data were included and analyzed in the further bioinformatics data mining, indicating the correlation of CFHR4 expression with the clinicopathological characteristics of HCC.

### Data mining in GEO and TCGA-LIHC for HCC

The raw array data of four GEO microarray series and TCGA-LIHC were subjected to background correction, quartile data normalization, and converted into gene expression values. Data were normalized using the affy package of Bioconductor R package (https://cran.r-project.org/mirrors.html). The differentially expressed genes (DEGs) between HCC tumor and normal samples were identified in four GEO microarray series using the limma package. The *P*-value < 0.05 and |log FC|>2 were chosen as cut-off criteria. Volcano plot and Venn diagram of DEGs were performed with ggpubr (https://CRAN.R-project.org/package=ggpubr) and VennDiagram (https://CRAN.R-project.org/package=VennDiagram) packages of R. The edgeR packages were used to identify CFHR4 expression between tumor and normal samples. The Spearman coefficients of the DEGs and CFHR4 were calculated, while DEGs with *P*-value<0.05 were defined as CFHR4-correlated genes. Correlation Heatmap of CFHR4-correlated genes was performed by R ggcorrplot (https://CRAN.R-project.org/package=ggcorrplot) package. ClusterProfiler and GOplot packages are used to draw bubble charts.

### GO and KEGG enrichment analysis

Gene ontology (GO), including biological process (BP), cellular components (CC), and molecular function (MF), and Kyoto Encyclopedia of Genes and Genomes (KEGG) enrichment analysis, was performed to discover the potential mechanism of the DEGs.

### Genomic alteration analysis

Genomic alteration types, alteration frequency, and protein change in amino acids were analyzed by the cBioPortal database (http://cbioportal.org) to investigate the CFHR4 mutation in TCGA-LIHC (42, 53, 54).

### Survival analysis

To analyze the correlation between CFHR4 expression and clinical relevance, a Cox proportional hazards model was applied to identify independent predictive factors for prognosis (55). The affection of CFHR4 expression on the survival of LIHC patients was evaluated by Kaplan–Meier plotter (https://kmplot.com/analysis/index.php?p=service&cancer=liver_rnaseq) (42, 56). Taking the median by Kaplan-Meier plotter of CFHR4 expression value as the critical point, TCGA-LIHC patients were divided into low and high expression groups to analyze the correlation between CFHR4 expression and clinicopathological characteristics (5).

### Immune infiltration analysis

Using the TIMER database (http://timer.cistrome.org/) (57–59), this study analyzed the correlation between the expression of CFHR4 and many types of infiltration of immune cells in TCGA-LIHC patients. *P*-value < 0.05 was statistically significant.

### Patients and clinical specimens

The present study was approved by the Ethics Committee of Sino-German Biomedical Center of the Hubei University of Technology, written consent was obtained from each participant, and the study was performed following the ethical standards of the Declaration of Helsinki (41). 17 HCC patients (2 females, 15 males; age 52.71 ± 11.10 years) undergoing tumorectomy were recruited, and pairs of fresh samples of human HCC and corresponding paracancerous tissues (at least 2 cm away from the edge of the tumor) were obtained for immunohistochemistry (IHC) and Western Blotting analysis from the same hospital. The samples were stored at -80°C until use.

### Immunohistochemical assay

The expression of the CFHR4 protein was assessed by IHC. The tumor tissues excised during the operation were immediately placed in 10% formalin for fixation, followed by dehydration, paraffin embedding, and sectioning. Primary antibody of mouse anti-human CFHR4 (R&D Systems, Germany) was used at a dilution of 1:100. Secondary antibody HRP conjugated goat anti-mouse IgG was applied (Vector Laboratories Inc, US). DAB chromogen was applied on the tissue and incubated for 60 mins, and counterstained in Hematoxylin. Slides were mounted and imaged using Keyence BZ-X810E Microscope (Keyence Inc, Japan).

### Western Blotting assay

According to the manufacturer’s instructions, total protein was extracted from tissues using the Protein Extraction Kit (Beyotime Biotech, Shanghai, China). Protein lysates were separated by 10% SDS-PAGE and then transferred to a PVDF membrane blocked with 5% skim milk powder at room temperature for 1h. After the membranes were blocked, they were incubated with the primary antibodies at 4°C overnight including, and anti-GAPDH (Proteintech Group, China), anti-CFHR4 (R&D Systems, Germany). Subsequently, the membranes were incubated with horseradish peroxidase-conjugated antibodies at room temperature for 1hr. Protein signals were detected using enhanced chemiluminescence (LI-COR Biosciences, USA). The results of Western Blotting were evaluated by densitometric analysis using ImageJ software.

### Quantitative reverse transcription PCR

Tissue biopsies obtained from HCC patient were weighted and homogenized by sonication in Qiazol Lysis Reagent (Qiagen, Hilden, Germany). RNA was isolated using chloroform extraction and dissolved in the TE buffer. The quantity and purity of RNA was evaluated using Nanodrop 2000 (ThermoFisher Scientific Inc., USA), and 1 μg of total RNA was reversely transcribed using HiScript Reverse Transcriptase (Vazyme Biotech Co. Ltd, China). qRT-PCR was performed in LightCycler 480 (Roche Diagnostics Ltd, Germany) using AceQ qPCR SYBR Green MasterMix (Vazyme Biotech Co. Ltd, China). Primers for the targeting gene *CFHR4* and housekeeping gene *ACTB* were listed below. Results were analyzed using dCt method.

**Table:** 

Gene	Forward (5′-3′)	Reverse (5′-3′)	Product Size (bp)
*CFHR4*, exon 2	CGCGTAGACCATACTTTCCA	ACCCATCTTGTGTGCAGTGA	117
*ACTB*	TTTTGGCTATACCCTACTGGCA	CTGCACAGTCGTCAGCATATC	109

### Statistical analysis

R software (version 3.5.1, The R Foundation), GraphPad Prism software (version 8.0, GraphPad Software, Inc.), and SPSS software (version 24.0, SPSS, Inc.) were applied for statistical analysis and scientific graphing. Student *t*-test (and reverse transcription PCR nonparametric tests) was used to compare the expression level of CFHR4 between HCC tumor and normal tissues (41); The Cox proportional hazards regression model was used to evaluate the hazard ratio (HR); The statistically significant parameters in univariate analysis were selected for Cox multivariate analysis and to confirm independent prognostic predictor factors (43). Kaplan-Meier and Log-rank tests were employed to identify the overall survival (OS) variables in HCC patients. The chi-square test was performed to determine the relationship between CFHR4 expression and the clinicopathological factors of HCC patients. *P*<0.05 indicates that the difference was statistically significant.

## Data availability statement

The original contributions presented in the study are included in the article/**Supplementary Material**. Further inquiries can be directed to the corresponding author.

## Ethics statement

The studies involving human participants were reviewed and approved by Ethical Review Committee of Life Science Sino-German Biomedical Center Hubei University of Technology (Official Seal). The patients/participants provided their written informed consent to participate in this study.

## Author contributions

KH, QY designed the study. QD, HL and ZX performed the study. QD and HL analyzed the results. KH, HL and QD wrote the manuscript. All authors contributed to the article and approved the submitted version.

## Funding

The study was supported to KH by the Initial Project for High Level Talents of HBUT, grant No. 337193, to HL by the Hubei Province Natural Science Foundation, grant No. 2021CFB289, and to QY by the National Natural Science Foundation of China, grant No. 81970548, as well as by the Medical Science Advancement Program (Clinical Medicine) of Wuhan University, grant No. TFLC2018003.

## Acknowledgments

We thank the patients and colleagues of the participating study, especially Dr. Dawei Huang from Department of Hepatology, Hubei Hospital of Traditional Chinese Medicine. We greatly appreciate the helpful and constructive comments from the reviewers during the preparation of the manuscript.

## Conflict of interest

The authors declare that the research was conducted in the absence of any commercial or financial relationships that could be construed as a potential conflict of interest.

## Publisher’s note

All claims expressed in this article are solely those of the authors and do not necessarily represent those of their affiliated organizations, or those of the publisher, the editors and the reviewers. Any product that may be evaluated in this article, or claim that may be made by its manufacturer, is not guaranteed or endorsed by the publisher.
